# Structures of Lysenin Reveal a Shared Evolutionary Origin for Pore-Forming Proteins And Its Mode of Sphingomyelin Recognition

**DOI:** 10.1016/j.str.2012.06.011

**Published:** 2012-09-05

**Authors:** Luigi De Colibus, Andreas F.-P. Sonnen, Keith J. Morris, C. Alistair Siebert, Patrizia Abrusci, Jürgen Plitzko, Vesna Hodnik, Matthias Leippe, Emanuela Volpi, Gregor Anderluh, Robert J.C. Gilbert

**Affiliations:** 1 Division of Structural Biology, Wellcome Trust Centre for Human Genetics, University of Oxford, Roosevelt Drive, Oxford OX3 7BN, UK; 2 Wellcome Trust Centre for Human Genetics, University of Oxford, Roosevelt Drive, Oxford OX3 7BN, UK; 3 Sir William Dunn School of Pathology, University of Oxford, South Parks Road, Oxford OX1 3RE, UK; 4 Department of Molecular Structural Biology, Max Planck Institute for Biochemistry, Am Klopferspitz 18, D-82152 Martinsried, Germany; 5 Department of Biology, Biotechnical Faculty, University of Ljubljana, Večna pot 111, 1000 Ljubljana, Slovenia; 6 Department of Zoophysiology, Zoological Institute, University of Kiel, Olshausenstr. 40, 24098 Kiel, Germany; 7 National Institute of Chemistry, Hajdrihova 19, 1000 Ljubljana, Slovenia

## Abstract

Pore-forming proteins insert from solution into membranes to create lesions, undergoing a structural rearrangement often accompanied by oligomerization. Lysenin, a pore-forming toxin from the earthworm *Eisenia fetida*, specifically interacts with sphingomyelin (SM) and may confer innate immunity against parasites by attacking their membranes to form pores. SM has important roles in cell membranes and lysenin is a popular SM-labeling reagent. The structure of lysenin suggests common ancestry with other pore-forming proteins from a diverse set of eukaryotes and prokaryotes. The complex with SM shows the mode of its recognition by a protein in which both the phosphocholine headgroup and one acyl tail are specifically bound. Lipid interaction studies and assays using viable target cells confirm the functional reliance of lysenin on this form of SM recognition.

## Highlights

► We report the structure of lysenin, a sphingomyelin (SM)-binding pore-forming protein ► Lysenin binds SM's headgroup and tail; the tail occupies the edge of a β sheet ► Lipid binding and live cell studies confirm activity dependence on SM recognition ► Lysenin is related to pore-forming proteins from disparate kingdoms

## Teaser

Lysenin is related to pore-forming proteins found in bacteria and fungi. De Colibus et al. describe structures of lysenin in different Apo conformations and bound to the lipid sphingomyelin. They map its evolutionary relationships and validate structural insights using affinity assays and live cell imaging.

## Introduction

Pore-forming proteins have evolved in all kingdoms of life, and are increasingly understood to exist in a limited number of superfamilies. The cholesterol-dependent cytolysins (CDCs) of Gram-positive bacteria, for instance, are structurally related to the membrane-attack complex/perforin (MACPF) family of proteins found in humans and *Plasmodium* (Amino et al., 2008; Anderluh and Lakey, 2008; Rosado et al., 2008). Another family is exemplified by aerolysin from *Aeromonas hydrophila* (Parker et al., 1994) and ε-toxin from *Clostridium perfringens* but includes also the fungal *Laetiporus sulphureus* lytic lectin (LSL) (Anderluh and Lakey, 2008; Cole et al., 2004; Mancheño et al., 2005). Thus, once evolved, the structure of individual domains, i.e., pore-forming modules (PFMs), seems remarkably well conserved. Although the amino acid sequence can change almost completely, the topology of the module remains preserved. In this study, we describe the structure of an additional member of the aerolysin family.

Commonly, pore-forming proteins engage a lipid or protein binding partner to recognize the target membrane. Subsequently they oligomerize on the surface of the bilayer and then insert into it to form a lesion. In this process, all pore-forming proteins must undergo a structural rearrangement to convert themselves from a soluble state to a membrane-inserted one (Anderluh and Lakey, 2008; Gilbert, 2010). This is frequently a remarkable transformation, such as the conversion of an α-helical structure in the soluble form of the protein to a β sheeted form in the membrane (Gilbert, 2005; Shatursky et al., 2000; Tilley et al., 2005), or vice versa (Mueller et al., 2009). The region that finally spans the membrane has consistently been found to be amphipathic in nature, in order to interface simultaneously with the aqueous pore and the hydrophobic acyl chains of the bilayer interior (Shatursky et al., 2000; Song et al., 1996). How proteins specifically bind to and recognize lipids is understood comparatively poorly, as only a small number of lipid:protein complex structures have been resolved. For example, lipids have been observed in a study of aquaporin-0 crystals: the path of the lipid chains across the surface of the protein was identified and found to be essentially determined by the acyl chain, irrespective of the lipid headgroup involved (Hite et al., 2010).

Lysenin from the earthworm *Eisenia fetida* is a pore-forming protein that specifically interacts with sphingomyelin (SM) and may confer innate immunity against parasites by attacking their membranes (Bruhn et al., 2006; Cooper et al., 2001). Lysenin has come to be valued as a label for SM, a sphingolipid critical for bilayer structure and function (Gault et al., 2010), in cell membranes (Hullin-Matsuda et al., 2009; Ishitsuka and Kobayashi, 2004). Studying the structure of lysenin bound to SM has the potential to reveal molecular details of the specific recognition of a lipid by a protein and to suggest a mechanism for the process of pore formation. Here we report the crystal structure of lysenin alone, and in complex with the sphingomyelin headgroup phosphocholine (POC), and with SM itself. The topology of the lysenin structural fold establishes it as a member of the aerolysin family of pore-forming proteins (Szczesny et al., 2011), which appears thus to be conserved from bacteria to annelids. The complex with SM shows how lysenin recognizes SM at full stretch, binding both its POC headgroup and its acyl tail. The headgroup is bound electrostatically but the tail is bound by ring-stacking-like interactions involving two critical tyrosine residues. We also find an additional POC-binding site, which indicates how lysenin might be guided in its attack on the target membrane. The SM-bound structure suggests that specific residues are involved in recognition of the lipid and by site-directed mutagenesis we confirm their importance using lipid binding assays and live cell imaging of target cells.

## Results

### Overall Structure of Lysenin

**Figure 1:**
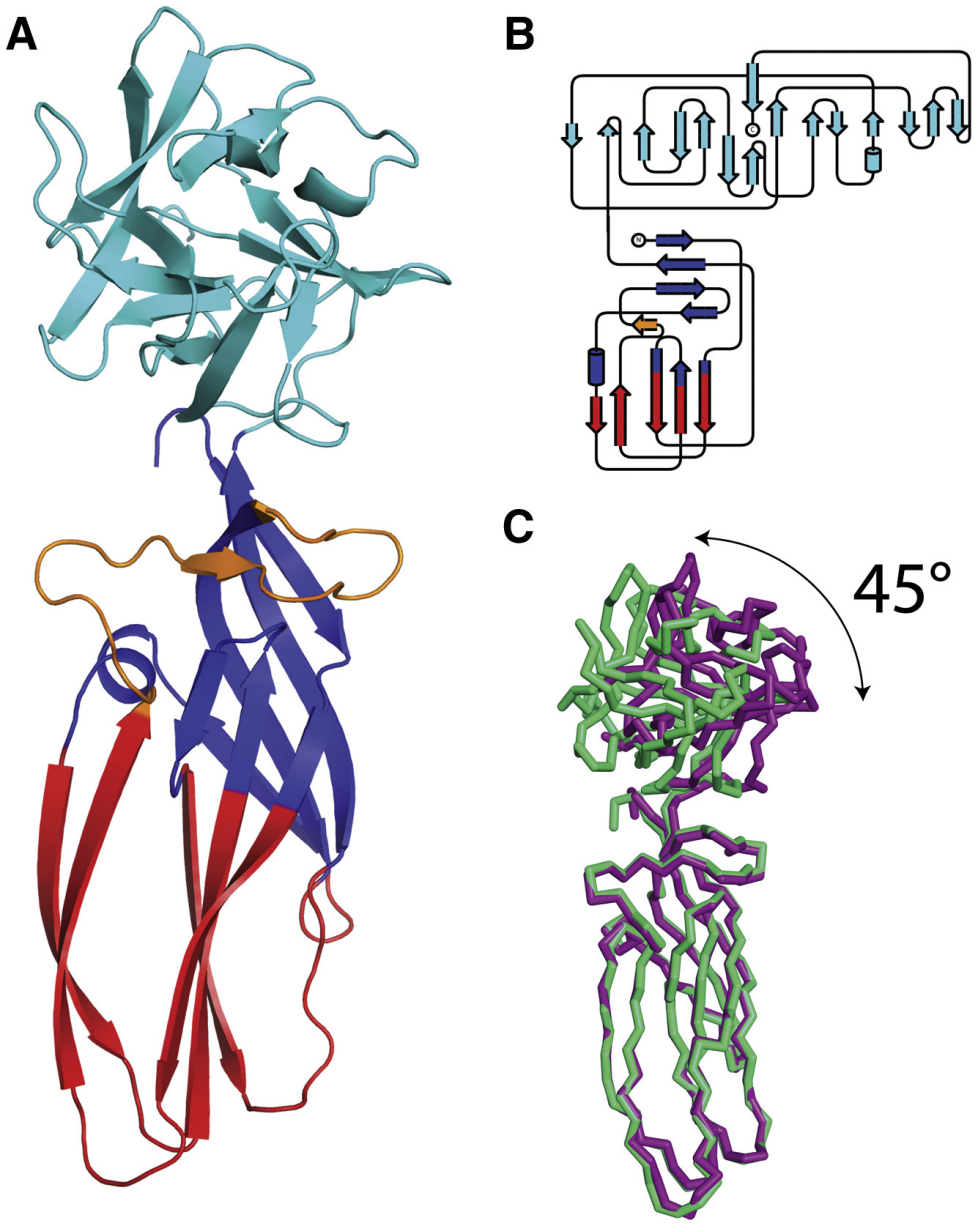
Lysenin Crystal Structure (A) Cartoon representation of the lysenin crystal structure shows it is organized into two domains: the PFM domain at the N-terminus made of subdomain 1 (red), subdomain 2 (blue) and the β-hairpin (orange); the β-trefoil C-terminal domain colored in cyan. (B) Lysenin protomer topology diagram. Color code is the one adopted in (A). (C) Superpositions of apo and POC-bound form of lysenin, respectively in violet and green. Superposing the PFM domains shows the alternative orientations of the head with respect to the N-terminal domain.

The crystal structure of lysenin was first determined in space group P6_5_22, with one molecule per asymmetric unit (a.u.), by multiple isomorphous replacement with anomalous scattering (MIRAS, one SeMet and one Hg derivative) and then in space group P1 with four molecules per a.u. by molecular replacement. The structure reveals that lysenin has two domains. The elongated N-terminal domain consists of a 3_10_ helix and 10 β strands, six of which belong to a highly twisted antiparallel β sheet (Figures 1A and 1B). The N-terminal domain can be divided into two subdomains; subdomain 1 has a β sandwich formed by a two- and a three-stranded antiparallel β sheet. Subdomain 2 consists of a double-turn 3_10_ helix, a β sandwich formed by a three- and four-stranded antiparallel β sheet and a β-hairpin within an additional long loop. The C terminus of lysenin is composed of a β-trefoil motif with a six-stranded antiparallel β-barrel capped on one end by three two-stranded hairpins and a single-turn 3_10_ helix (Figures 1A and 1B). The five crystallographically independent copies of the molecule (see Experimental Procedures and Table 1), define an ∼45° arc that is subtended by the C-terminal domains hinging at residues 159–168 (Figure 1C).

### Similarity to Pore-Forming Toxins of Known Structure

**Figure 2:**
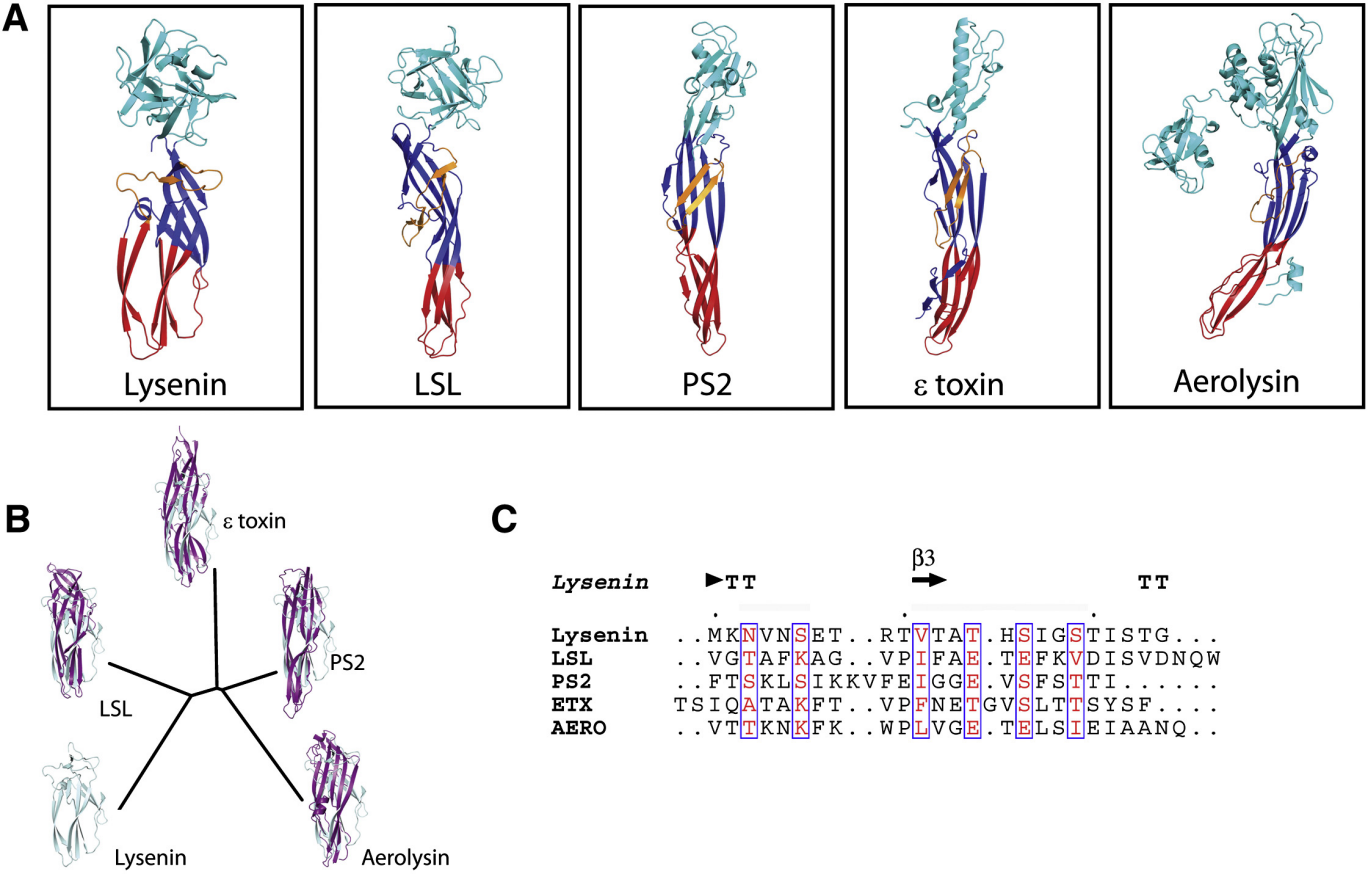
Conservation of the PFM across Homologs (A) Gallery of lysenin homologs identified by DALI (http://ekhidna.biocenter.helsinki.fi/dali_server), using the lysenin PFM as probe. (B) Structural phylogenetic tree expressing the evolutionary distance as rmsd of superposed PFM domains: lysenin-LSL, 1.65 Å; lysenin-ε toxin, 1.87 Å; lysenin-aerolysin, 1.88 Å; lysenin-parasporin2, 1.65 Å. (C) Multiple sequence alignment of the putative transmembrane hairpin form homologs described in (A) shows that alternating pattern of hydrophobic/hydrophilic residues despite the very low sequence conservation. Alignment was built using CLUSTAW and the figure was prepared with ESPript2.2 server (http://espript.ibcp.fr/ESPript/ESPript/). See also Figure S1.

The N terminus of lysenin is immediately reminiscent of other pore-forming proteins. Using the lysenin N-terminal domain as a probe in DALI (Holm and Rosenström, 2010), four structures with high similarity are found: the lytic lectin of the mushroom *Laetiporus sulphureus* (LSL) (1w3a-A, Z = 4.7, root-mean-square deviation [rmsd]_Cα_ = 4.9, α carbon positions aligned = 98/312 residues) (Mancheño et al., 2005), Gram-positive *Clostridium perfringens* ε-toxin (1uyj-B, Z = 4.5, rmsd_Cα_ = 4.1, α carbon positions aligned = 102/289 residues) (Cole et al., 2004), *Bacillus thuringiensis* parasporin-2 toxin (2ztb-A, Z = 4.5, rmsd_Cα_ = 3.7, α carbon positions aligned = 95/246 residues) (Akiba et al., 2009), and aerolysin from the heterotrophic Gram-negative bacterium *Aeromonas hydrophila* (3g4o-A, Z = 3.6, rmsd_Cα_ = 4.7, α carbon positions aligned = 98/460 residues) (Iacovache et al., 2011) (Figure 2A). This finding indicates that the N terminus is indeed the pore-forming module (PFM) of lysenin. Notably, the lysenin PFM aligns with the functionally equivalent regions of homologous proteins, such as the PFMs of ε-toxin and parasporin (Cole et al., 2004; Akiba et al., 2009). Thus, although lysenin is an annelid protein, it is clearly evolutionarily related to a family of pore-forming proteins found in prokaryotes, as shown in the structural phylogenetic tree (Figure 2B). Interestingly, although both eukaryotic, lysenin, and LSL lie on independent branches of the phylogenetic tree and as distant from each other as they are from the prokaryotic family members. The structural comparisons made here are in agreement with a recent bioinformatics analysis (Szczesny et al., 2011) that argued that lysenin belongs to the extended aerolysin family characterized by a conserved core β sheet structure elaborated by insertions specific to each family member. For example lysenin has an additional β-hairpin and a double-turn 3_10_ helix after the so-called “insertion loop,” which like those of related proteins displays an alternating pattern of hydrophobic and hydrophilic residues running from residues Met44 to Gly67 (subdomain 2) (Figure 2C). This patterning is typical for regions of pore-forming proteins undergoing a refolding transition from a solution state to a membrane-inserted state and suggests that the bilayer-penetrating region of the structure makes use of this region (Shatursky et al., 2000; Song et al., 1996). Members of the aerolysin family contain a surface patch of Ser and Thr residues that may have a role in oligomerization or in positioning of the protein on the membrane before membrane insertion (Akiba et al., 2009). This feature is also found in the PFM of lysenin (Figure S1 available online).

### Phosphocholine Binding Pocket

**Figure 3:**
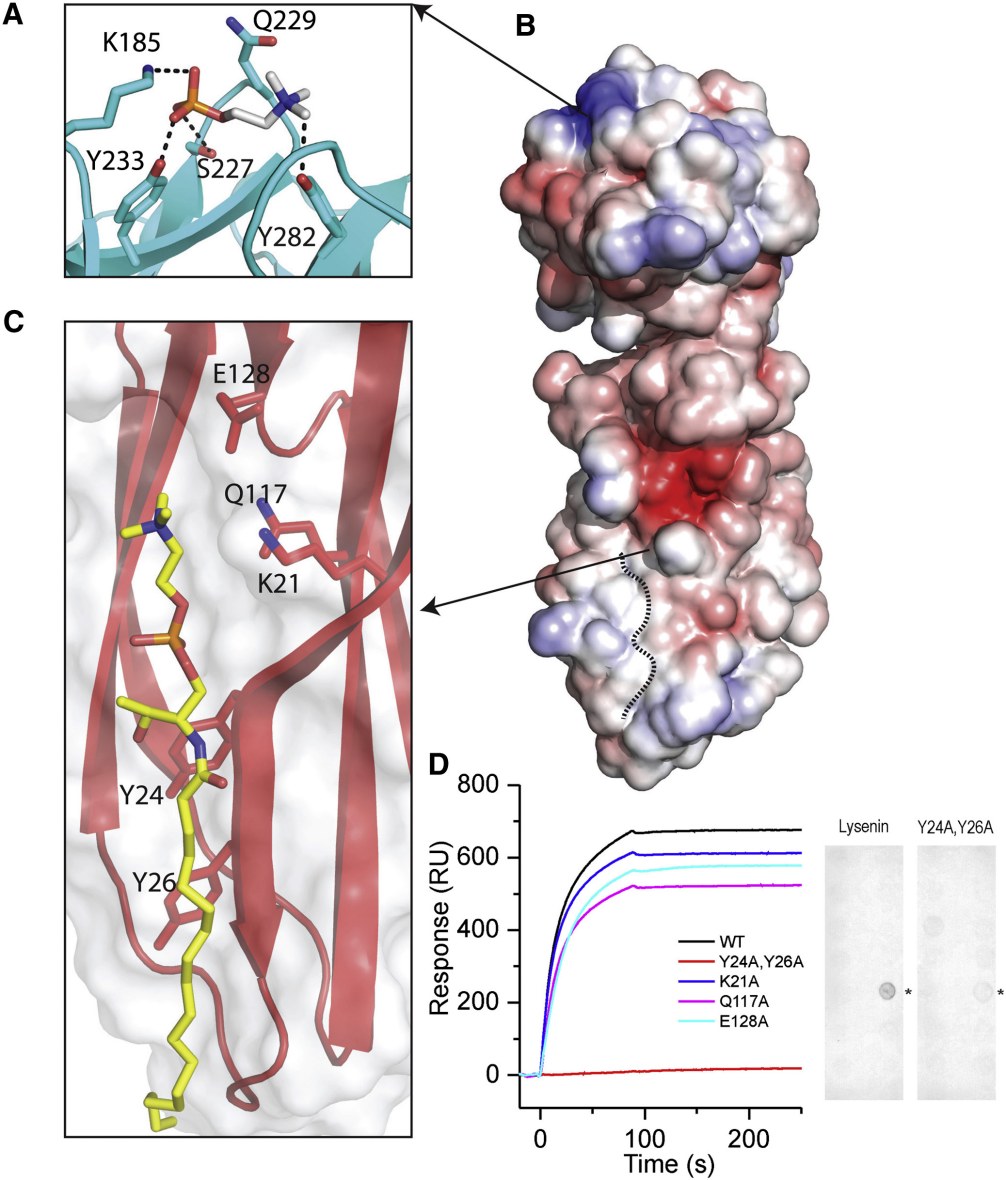
Molecular Details of POC and SM Binding Sites (A) Close-up of the POC-binding region within the C-terminal head domain. Residues directly involved in the binding are shown as sticks and hydrogen bond and electrostatic interaction are shown as dash lines. (B) Electrostatic surface representation of lysenin within an energy range from −5 to +5 kT, respectively from red to blue. The dotted line highlights the groove where SM is bound at the edge of the β sheet. (C) Close-up of the PFM with SM bound to it. Lysenin is represented as cartoon and surface. Residues involved in interaction with SM are represented as sticks. SM is represented as stick and colored in yellow. (D) Comparison of SM binding properties of lysenin WT and its mutants probed by SPR. Overlaid sensograms previously described lysenin variants show that the double mutant Y24A-Y26A is unable to interact productively with SM. Dot-blot, as inset, confirms that WT lysenin is able to bind selectively SM but double mutation Y24AY26A abrogate the binding. ^∗^Indicates position of SM dot. See also Figure S2 and Movies S1 and S2.

In the crystal form with space group P6_5_22 the protein was found in complex with a phosphocholine molecule (lysenin:POC) (Figure 3A). This lipid headgroup binds specifically within the β-trefoil via a hydrogen bond network involving residues Tyr233, Ser227, Tyr282, and a salt bridge interaction with Lys185. Residue Gln229 is the gate regulating access to the POC binding pocket (Figure 3A): in the apo forms of lysenin, Gln229 is in a closed conformation, blocking the entrance to the POC binding site, but has opened in the POC bound state (see Movie S1). Lys185 aids in defining the gate, as it is in a different conformer in the apo form, preventing interaction with the phosphate moiety. Unlike other β sheeted POC-binding proteins, such as staphylococcal LukF, the POC pocket of lysenin does not involve cation-π interactions between the electron-rich systems of the tyrosine aromatic rings (Olson et al., 1999), but it is similar to that found in actinoporins (Mancheño et al., 2003). A global analysis of the lysenin surface electrostatic potential reveals that there is a greater concentration of positive charges in the lysenin β-trefoil C terminus than in the N-terminal PFM (Figure 3B).

### Structure of Lysenin Bound to Sphingomyelin

The crystal structure of lysenin bound to sphingomyelin (SM) was determined in the spacegroup C2 by molecular replacement. To our knowledge, this is the first crystal structure of SM bound to a protein and it shows simultaneous recognition of hydrophilic and hydrophobic portions of the lipid. A binding site for the lipid head group at the top of the PFM is partnered by interaction with one of the two hydrophobic tails of a pair of aromatic residues on the edge of a β sheet (Figure 3C) (see Figure S2 for an omit map; the other SM tail appears to be disordered). A long groove along the edge of the PFM then contains the SM tail (Figure 3C). The crystals exhibited two molecules in the asymmetric unit, one bound to SM and one not. The SM-bound copy has a different conformation in subdomain 1 of the PFM to the protein structure without SM bound, because it has opened up into a more loosely packed arrangement (see also Movie S2). The SM binding site is the most striking feature, because the fatty acid moiety of the lipid flanks the edge β strand 2 of the PFM the way an additional polypeptide strand would. Residues Glu128, Gln117, and Lys21 interact with the SM head group, making a hydrophilic binding site, and the main-chain CO group of Gly23 interacts with the phosphate moiety at the SM head by hydrogen bonding. The single SM hydrophobic tail resolved is held nearly parallel to a β strand of the PFM by the side chains of Tyr24 and Tyr26, which form van der Waals interactions with the SM tail, creating a sort of aromatic platform to hold the tail in place. However, the SM:lysenin complex seems to be in a pre-pore state, because it has not yet undergone reconfiguration or oligomerized to a membrane-inserted form.

### Correlation between SM Binding Ability and Lytic Activity of Lysenin

To confirm the authenticity of the SM binding site shown in our SM:lysenin crystal structure, we carried out alanine site-directed mutagenesis of Lys21, Gln117, Glu128, and generated a double alanine mutant of Tyr24 and Tyr26. All the lysenin variants were successfully purified and produced fluorescence spectra comparable to the WT protein (Figure S3). However, surface plasmon resonance (SPR) experiments on the lysenin mutants reveal that mutation of Lys21, Glu128, and Gln117 reduce SM binding (Figure 3D), whereas the double tyrosine mutant almost abolishes SM binding ability, as predictable on the base of our SM:lysenin crystal structure. This is also supported by a lipid dot-blot analysis showing SM binding by WT protein but not by the double tyrosine mutant (Figure 3D). Previously published work (Kiyokawa et al., 2004; Kwiatkowska et al., 2007) has described how the mutation Trp20Ala in the PFM of lysenin somewhat reduces SM binding and renders lysenin unable to oligomerize and lytically inactive. Our structure shows that the Trp20Ala mutant does not directly interact with the SM but ensures correct orientation of the SM-interacting Lys21 and stabilizes the PFM fold.

To ensure that the effects we observe for the Tyr24Ala-Tyr26Ala double mutant are specific to those residues and not the result of a unfolding of the PFM when these mutations are performed, we made a direct comparison of molecular dynamics of wild-type and double mutant lysenin. As shown in Figure S4, the dynamic fluctuation of the wild-type and double mutant proteins is the same over a 10 ns period. This shows that there has been no long range alteration in the lysenin structure when the mutations are present and that the effects observed are specific for the absence of the two tyrosines against which the SM tail aligns.

**Figure 4:**
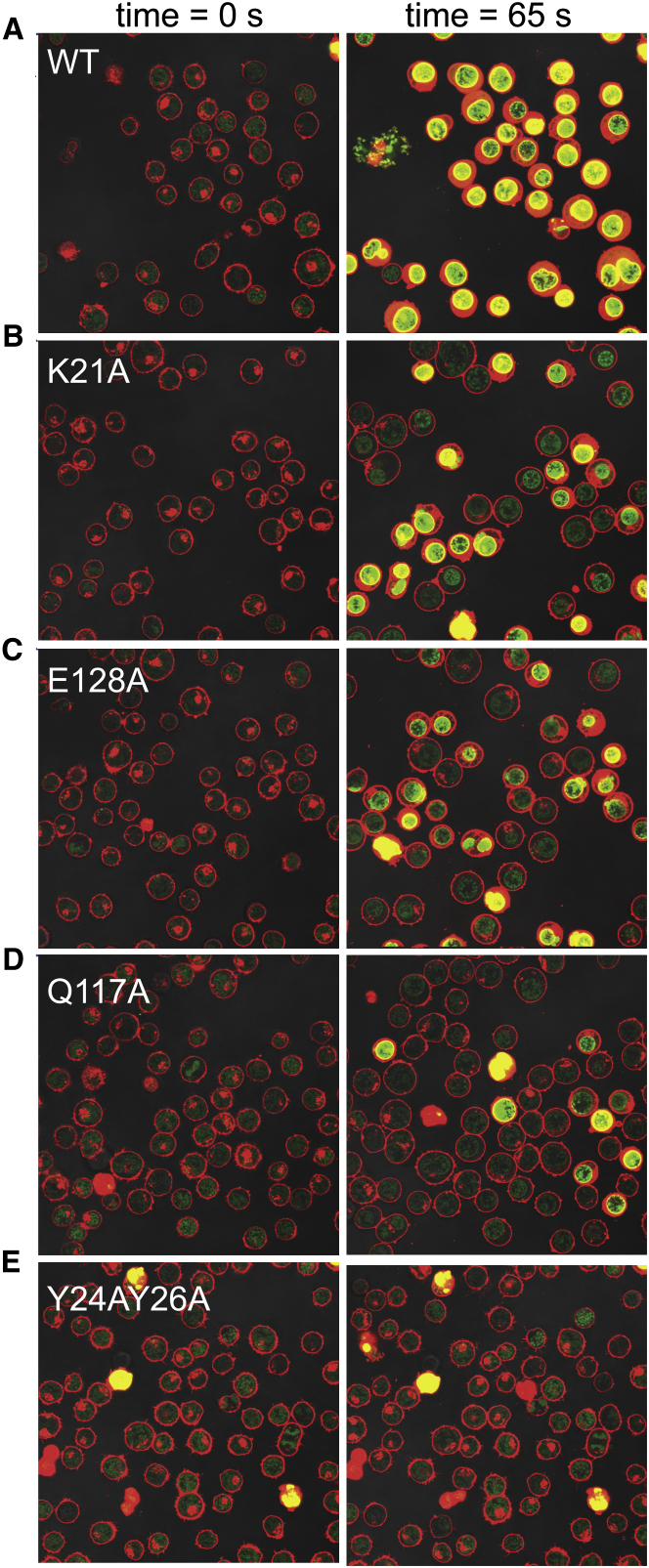
Live Cell Imaging of Wild-Type and Mutant Forms of Lysenin (A) Confocal laser scanning microscopy (CLSM) images of cells with WT lysenin at ∼1 μg/ml and at time zero and 65 s. See Movie S3 for image sequence. (B) As (A) for the mutant Lys21Ala but see Movie S4. (C) As (A) for the mutant Glu128Ala but see Movie S5. (D) As (A) for the mutant Gln117Ala but see Movie S6. (E) As (A) for the double mutant Tyr24Ala Tyr26Ala but see Movie S7. See also Figures S3 and S4.

We also sought to assess the lytic effect of lysenin and its mutants on Jurkat cells, monitoring pore formation on the plasma membrane, and subsequent cell death, using the membrane-impermeable nucleic acid-binding fluorescent dye SYTOX Green. These experiments were performed by taking time-lapse images over a timeframe of 65 min after addition of lysenin. The pore-forming effects of the proteins correlate well with the extent to which lysenin is able to bind SM. In fact, wild-type (WT) lysenin (Figure 4A and Movie S3) causes the cells to become SYTOX Green bright within 7 min, whereas the impaired SM binding variants (Lys21Ala, Gln117Ala, Glu128Ala) produce a comparable effect between 10 and 13 min (Figures 4B–4D and Movies S4–S6). As expected, the double mutant Tyr24Ala Tyr26Ala (Figure 4E and Movie S7) has very low pore-forming activity, with dye permeation completed only in ∼30 min in a first damaged cell, whereas at the end of the time lapse period an observable toxic effect could be visualized in just one-third of the cells. Taken altogether, SPR, dot-blot, molecular dynamics, and microscopy data confirm that in vivo SM binding occurs as shown by our SM:lysenin structural model and that the mutated residues are crucial for engagement of lysenin with both the SM headgroup and acyl tail.

### Oligomers of Lysenin in the Presence of SM

**Figure 5:**
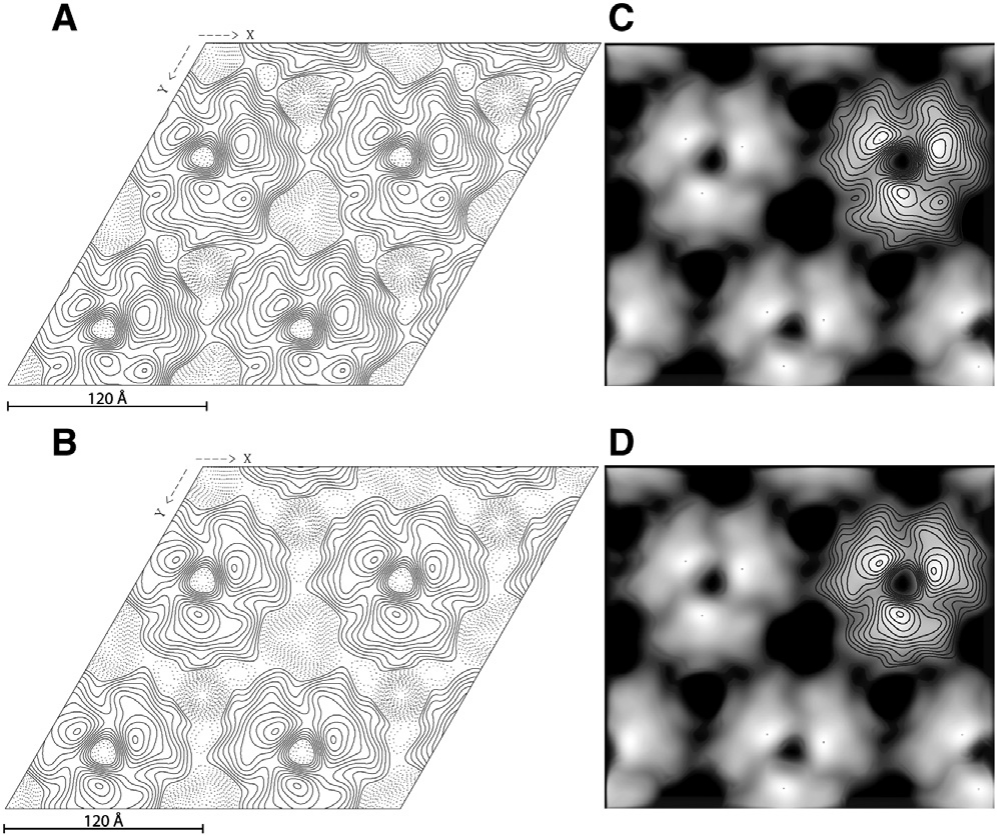
Two-Dimensional Electron Microscopy of the Lysenin Pore (A) Projection map of the crystal in *p*1 space group. (B) Projection map of the crystal in *p*3 space group. (C) Thresholded projection of the 2D structure at 14 Å with the contour map in *p*1 space group superimposed. (D) Thresholded projection of the 2D structure at 14 Å with the contour map in *p*3 space group superimposed. Symmetry imposition does not introduce any major changes in the features of the projection map. See also Figure S5.

Lysenin was previously observed to produce a characteristic oligomeric honeycomb structure in SM-containing membranes by negative stain electron microscopy (Kwiatkowska et al., 2007; Yamaji-Hasegawa et al., 2003). However, the lack of structural data on lysenin oligomers makes it difficult to elucidate the mechanism of pore formation despite our structural insights into the isolated protein. We therefore thought to image lysenin in a lipid bilayer (Figure S5) by 2D electron crystallography and found that it generates a putative oligomeric pore placed in a honeycomb array as previously observed (Figure 5). This crystal is a trigonal array with space group *p*3 (Table 2). We calculated an electron density map from which we estimated that the diameter of the trimeric assembly measures up to 112 Å, with an inner pore of 57 Å (Figure 5). We are not able to model the lysenin oligomeric assembly in more detail because we do not know the major structural reconfiguration undergone by the protein upon membrane interaction. However, the overall shape of the asymmetric unit in the trimeric lattice suggests a side-on alignment of the protein in the membrane, with pore-forming regions penetrating the membrane below.

## Discussion

In this study, we have described the structure of lysenin, a sphingomyelin-specific pore-forming protein, in its apo form and bound to POC and SM. By structural analysis we have shown that lysenin is related to pore-forming proteins from across the biosphere. It is particularly striking that the homologous N- and C-terminal domains of fungal LSL (Mancheño et al., 2005) and annelid lysenin are in alternative positions in their respective sequences. Therefore, during their divergent evolution from a common ancestral protein, there has most likely been a genetic swap of the two domains with respect to each other. In addition, the SM-binding region of lysenin is an added edge to the PFM sheet compared to other pore-forming proteins.

We suggest that lysenin interacts with the membrane initially by binding of POC in lipids such as SM through its β-trefoil, after attraction to the membrane surface through charge-charge affinity; it will then bind the full length of SM. The insight that lysenin interacts with membranes in a two-stage process is strengthened by previously published work: removal of the β-trefoil domain reduces the protein’s affinity 100-fold (Kiyokawa et al., 2005), showing that the C-terminal domain is needed in membrane binding even though it is the N-terminal PFM that binds SM specifically. The positively-charged patch on the C-terminal domain would make for interactions with negative charges at the membrane surface, as for example found at in the case of the sulphates in proteoglycans such as heparin sulphate and chondroitin sulphate, and may help in attracting or guiding the approach of lysenin to the membrane surface.

The structure of the SM/lysenin molecular complex represents, to our knowledge, the first crystal structure showing a direct and specific SM/protein contact, where not only the headgroup but also the acyl chain of the lipid is recognized simultaneously. The interaction with the acyl chains of SM by lysenin was already shown by differential scanning calorimetry experiments (Yamaji-Hasegawa et al., 2003). The SM/protein complex is also in agreement with recently published results on the molecular recognition of sphingolipids by the protein transmembrane domain (TMD) of COPI (coat protein) machinery protein p24 (Contreras et al., 2012) where Förster resonance energy transfer (FRET), alanine scanning and molecular dynamic demonstrated a direct and highly specific interaction of sphingomyelin species with the TMD. Strikingly the interaction depends on both the head-group and the backbone of the sphingolipid, as in our structure, and on the presence of a signature sequence (VXXTLXXIY) within the TMD. The acyl chain of SM appears to pack in the groove between Val13, Thr16, and Leu17 of the p24 TMD. One acyl chain of SM also occupies a groove on the lysenin PFM domain directly interacting with tyrosines 24 and 26, although it is defined by β strands and not as in p24 α helices.

To date, interaction with SM has been documented in actinoporins, which are SM-dependent pore-forming toxins from sea anemones, only via the SM headgroup and chemical moieties immediately beneath it (Mancheño et al., 2003; Bakrac et al., 2008). SM occupies a similar position to the lipids found in the aquaporin-0 2D crystal structure (Hite et al., 2010), and Kir2.2 potassium channel (Hansen et al., 2011). Thus, like the cholesterol-dependent cytolysins (CDCs), in lysenin specific lipid/protein interactions lead to membrane disruption as a function of both the protein inserting into the membrane and the lipid reorganization induced (Gilbert, 2010). We suggest that binding of SM in one leaflet of the targeted membrane would result in its reconfiguration during oligomerization of lysenin, to disrupt the membrane and form a pore. In this way, by directly binding to a particular lipid component of the membrane, lysenin can be specifically targeted and can couple oligomerization to both its own refolding and the reorganization of the membrane. The specific binding of SM over its whole length would give oligomerizing lysenin sufficient purchase to disrupt the energetically stable lipid bilayer.

In order to investigate lysenin in its oligomeric state, we collected electron crystallography images of lysenin in liposomes containing SM. These data allowed us to identify the trigonal symmetry of the oligomer and estimate its dimensions. Lysenin pores are known to be small, with an approximate hydrodynamic diameter of 3 nm (Yamaji-Hasegawa et al., 2003). The trigonal lattice contains a well-defined trimeric unit, in which the lysenin monomers appear to lie flat. The shape of the protomeric unit in this lattice does not look the same as lysenin in any projection and we cannot say therefore whether they are monomers or dimers of the protein. The absence of any extensive hydrophobic regions on lysenin’s surface, as found in other pore-forming proteins, means that it is likely similarly to deploy a β-hairpin across the membrane. A lysenin trimer could only supply three hairpins, or six β strands that is not enough to form a β-barrel and in itself suggests that the pore forming state may be a hexamer (a trimer of dimers). We believe that the structure observed in our 2D crystals is in fact a pre-pore state. The structures described in this study suggest that lysenin interacts with POC—not necessarily only that of SM but also from phosphocholine lipids—via its lectin domain prior to pre-pore assembly and pore formation. Subsequently, it associates with the head group and one full-length aliphatic tail of the SM molecule, which would serve to deform the membrane bilayer on the path to pore formation. Overall, our structures provide a rationale for the further development of lysenin as a tool for studying the role of SM in membrane structure, dynamics and function, while explaining the molecular basis of its dependence on SM binding for full activity (Bruhn et al., 2006).

## Experimental Procedures

### Recombinant Protein Production and Purification

cDNA sequences encoding full-length lysenin were cloned into pTet (Invitrogen) in frame with the coding sequence for the thrombin cleavage site and for six histidine residues at the C terminus of the protein (LVPRGSGHHHHHH). The lysenin expression plasmid was termed pTetLys1 (Bruhn et al., 2006) and checked by sequencing for fidelity. Mutations were introduced into the pTetLys1 by site direct mutagenesis using the XL QuickChange kit (Agilent). Derivative plasmids were verified by DNA sequencing.

For expression of lysenin, pTetLys1 was transformed into *Escherichia coli* strain BL21. The transformed cells were grown in Terrific Broth medium at 22°C, with the addition of 100 μg/ml ampicillin in the medium. Identical conditions were adopted to express all lysenin mutated variants. Protein expression was induced with 0.2 μM anhydro tetracycline when the optical density at 600 nm reached 0.8. After 16 hr, cells were harvested, washed in PBS and stored at −80°C for later use.

For expression of SeMet-labeled protein, B834(DE3) cells were transformed with pTetLys1 plasmid. Cells were inoculated in Seleno-Met media (Molecular Dimensions) (Ramakrishnan et al., 1993) supplemented 100 μg/ml ampicillin and selenomethionine according to recommendations of the supplier. Protein expression was induced as described above.

Cells were lysed in phosphate-buffered saline (PBS) (137 mM NaCl, 2.7 mM KCl, 100 mM Na_2_HPO_4_ 2 mM KH_2_PO_4_, pH 7.4) supplemented with protease inhibitors (Complete without EDTA, Roche) by a cell disruptor. The clarified lysate containing the soluble recombinant protein was subjected to immobilized metal affinity chromatography (IMAC) onto an Ni^2+^-nitrilotriacetic acid-agarose column (Qiagen), followed by size-exclusion chromatography (SEC) on a HiLoad Superdex 200 pg 16/60 (GE Healthcare). The protein was eluted in 20 mM HEPES, 150 mM NaCl, 1 mM β-mercaptoethanol.

The purified native protein was tested for oligomerization in presence of 900 nmol β-octylglucopyranoside and 900 nmol sphingomyelin (brain, porcine; Avanti Polar Lipids). The rest of the purified protein was used in crystallization trials.

### Crystallization and Data Collection

Native lysenin and selenomethionine-labeled (Se-Met) lysenin were concentrated to 10 mg/ml and 200 nl sitting drops were set up using a 1:1 ratio between protein and precipitant in 96-well Greiner crystallization plates using a Cartesian dispensing robot (Walter et al., 2005). All crystals were unit soaked in the mother liquor plus 25% of glycerol as cryosolvent, followed by flash freezing in liquid nitrogen.

Hexagonal crystals of native lysenin grown in 1.8 M ammonium di-hydrogen phosphate, 1.0 M sodium acetate pH 4.5 were derivatized by a 45 min soak in a saturated solution of ethyl mercury phosphate (EMP). A single wavelength data set (SAD), was collected to 2.54 Å resolution at the Hg L-III absorption edge on beamline BM-14. These crystals belong to space group P6_5_22 with one molecule in the asymmetric unit.

Hexagonal crystals of Se-Met protein grew in the same crystal form in 1.8 M ammonium di-hydrogen phosphate, 1.0 M sodium acetate pH 4.5. A three-wavelength anomalous diffraction (MAD) data set with a resolution limit of 2.8 Å was collected at ESRF on beamline BM-14. These crystals before freezing were soaked in mother liquor and 50 mM phosphocholine (POC).

Triclinic crystals of native lysenin grew at 20°C in 1 M MES, 1.6 M magnesium sulfate pH 6.5. A 3.3 Å native data set was collected at ESRF on beamline ID14EH3. These crystals belong to space group P1 with four molecules in the asymmetric unit.

The sphingomyelin:lysenin complex crystals grew from the 1:1 molar mix (see above) in 0.1 M Bis-Tris pH 5.5, 0.2 M Li_2_SO_4_. The sphingomyelin:lysenin crystal diffracted to 3.1 Å at the Diamond Light Source on beamline I04. These crystals belong to space group C2 with two molecules in the asymmetric unit.

### Structure Determination

The indexing and integration of all data sets was carried out with XDS (Kabsch, 1993), and CCP4 (Collaborative Computational Project, Number 4, 1994) programs SCALA (Evans, 2006) and TRUNCATE (French and Wilson, 1978) were used for scaling and merging of diffraction data and calculation of structural factor amplitudes. All model building was done in Coot (Emsley and Cowtan, 2004).

The hexagonal Se-Met and Hg derivative crystals were highly radiation damage sensitive, and the data processing was performed by selecting the best frames.

Hg sites were located by SHELXD (Schneider and Sheldrick, 2002) using the Hg-SAD data collected at the L-III absorption edge from a crystal of the hexagonal crystal form. The heavy atom substructure was refined and initial phases were calculated with the program SHARP (de la Fortelle and Bricogne, 1997). The Se atom positions were located in an anomalous Log-likelihood gradient map and the Hg and Se substructures were refined and used for phasing in SHARP, using the native amplitudes in a two-derivative MIRAS calculation. Density modification in SOLOMON (Abrahams and Leslie, 1996) produced an interpretable electron density map, the C-terminal domain being especially easily traceable. The preliminary model was built by Buccaneer (Cowtan, 2006) and manually completed and corrected in COOT.

This preliminary model was used for molecular replacement with the program PHASER (McCoy et al., 2007) against the amplitudes of the triclinic crystal form, locating the first three molecules in the asymmetric unit. The fourth molecule’s N-terminal domain was then located in a separate search in Phaser, whereas the C-terminal domain was located in the initial F_o_-F_c_ type map using the spherically averaged translation function (SAPTF) (Vagin and Isupov, 2001) as implemented in MOLREP.

The triclinic crystals have well defined density for the N-terminal domain. The model was therefore completed in the triclinic crystal form. To improve the electron density of the N-terminal domain in the hexagonal crystal form, multi-crystal averaging was performed in CCP4-DMMULTI (Cowtan, 1994), with 5-fold averaging between the native triclinic crystal form (four copies/asymmetric unit, model phases) and the hexagonal SeMet crystal form (one copy in the asymmetric unit, MIRAS phases). Separate averaging masks were used for the N-and C-terminal domains. Despite the high resolution of the Hg data set the anisotropy of the data was very high and even after anisotropy correction it was not possible to see clear density for the N-terminal domain. For this reason no refinement was carried out on this crystal form.

The Se-Met refined structure was the one used in PHASER to obtain a molecular replacement solution for the lysenin-sphingomyelin complex. An omit map was calculated demonstrating presence of the bound lipid using a combination of CNS 1.3 (Brünger et al., 1998) and auto-BUSTER-TNT 1.11.1 (Blanc et al., 2004).

All structures were refined by auto-BUSTER-TNT 1.11.1 (Blanc et al., 2004). A TLSMD (Painter and Merritt, 2006) analysis was used to define nine TLS groups for the Se-Met crystal structure (295 residues), six TLS for each protomer, except the protomer D refined with five TLS, in the native crystal structure (chain A, 292 residues; chain B, 293 residues; chain C, 289 residues; chain D, 288 residues). Seven TLS were used for protomer A (292 residues) and five for protomer B (292 residues) in the lysenin sphingomyelin complex. The structures were validated with MOLPROBITY (Chen et al., 2010). Molecular graphics representations were created using the software PyMOL (http://www.pymol.org).

### Molecular Dynamics Simulations

The systems were energy minimized and equilibrated for 1 ns with 2 Kcal/mol/Å^2^ positional restraints, after which the restraints weight was increased to 5 Kcal/mol/Å^2^ and production simulations run for 10 ns on each system, with 8 Å cutoffs and PME used for long-range electrostatics (Essmann et al., 1995). Both the simulations and data analysis were preformed with AMBER11(Case et al., 2005).

### Surface Plasmon Resonance

SPR binding experiments were conducted by using a Biacore X instrument (GE Healthcare, Biacore) as described elsewhere (Anderluh et al., 2005). The first flow cell of the L1 sensor chip was covered by 1,200 RU of 1-palmitoyl-2-oleoyl-*sn*-glycero-3-phosphocholine (POPC) large unilamellar vesicles (LUV) of 100 nm in diameter and the second flow cell was covered by the same amount of POPC/sphingomyelin 9/1 (mol/mol) LUV. The concentration of the proteins was 200 nM in running buffer composed of 10 mM HEPES, 150 mM NaCl, pH 7.4.

### Lipid Dot Blots

The lipid dot blot experiment were performed exactly as described in (Bakrac et al., 2008) by using 2.5 μg/ml of protein to blot the lipids on SphingoStrips (Echelon Biosciences).

### Electron Microscopy

Images of 2D crystals were assessed and processed with the 2DX software package (Gipson et al., 2007). Crystal images exhibiting isotropic diffraction to beyond 20 Å were processed by correlation averaging with subsequent unbending, as implemented in the MRC software suite (Crowther et al., 1996). The crystal images plane groups of symmetry were assessed by phase comparison using Allspace (Valpuesta et al., 1994). *P*3 symmetry and subsets thereof were indicated. Four images were merged onto a common *p*3 phase origin after multiple search and refine iterations in 2DX. The overall phase residual was calculated in *p*3 to be 45° at a resolution of 14 Å. Images were contoured at 0.2× the SD in the density with solid contours (that represent protein) for density greater than −20.

### Construction of Structural Phylogeny

Superimposition of all PFMs was performed using SHP (Stuart et al., 1979) as previously reported (Riffel et al., 2002). The phylogenetic tree was calculated using a pairwise evolutionary distance matrix determined from the superimposed domains. The tree representation was generated using the programs FITCH and DRAWTREE as part of the PHYLIP package (Felsenstein, 1997). Structural comparisons were displayed using PyMOL.

### Live Cell Imaging

Jurkat cells (Schneider et al., 1977) were cultured in 75 ml flasks with RPMI, 10% FCS, 1.5% L-glutamine and 1.5% penicillin and streptomycin. A few days prior to imaging the cell cultures were passaged by 15% of the primary cell culture being transferred to new media, so that the cells could be imaged nearer a log growth phase. Prior to imaging 1 ml of the cell culture was washed three times in HBS buffer (20 mM HEPES, 150 mM NaCl, pH 7.4), and spun down between washes at 1,000 rpm (170 × g) for 3 min. After removing the supernatant, the cells were placed in 1 ml of HBS and resuspended by pipette and 3 s on a vortex shaker. The cell suspension was transferred to a PAA Labs 3.5 cm confocal Petri dish, with the cell buffer filling the middle well, immediately above the glass coverslip. Approximately 0.01 μl of CellMask PM Orange (Invitrogen red fluorescence emission cell membrane marker) and 0.5 μl of Sytox Green (Invitrogen green fluorescence emission cell viability marker) were added to the HBS buffered cell culture a few minutes before imaging.

The PAA Labs Petri dish was immediately transferred to a Zeiss Pecon XL3 large stage incubator with Pecon Labtek heated insert, attached to an Axiovert 200M microscope on a Zeiss 510 MetaHead laser scanning confocal system. The stage incubator temperature was set to 37°C. HeNe laser 453 nm 1.2 mW laser excitation power was set to 5.0% and Argon laser 488 nm 30 mW laser excitation power set to 0.5%, in order to minimize light damage to the cells during imaging. After 5 min in the incubator chamber the Petri dish lid was removed and a time-lapse started, set to continuous DIC transmission and red/green fluorescence imaging, with one scan every 13 s, at 1,024 × 1,024 pixels, with 2× averaging applied. Time-lapse video images were acquired using a Zeiss 63× 1.4 NA Plan Apochromat DIC oil immersion objective set to 1× zoom (i.e., 630×) magnification.

In order to ensure thorough mixing of lysenin with the cells, it was initially prediluted in HBS and then 200 μl of that added to 200 μl cells again in HBS. Lysenin was added at ∼1 min into the time lapse sequence. Microscope images were acquired using LMS 510 v4.2 confocal software and the time-lapse videos were visualized/exported using Zeiss LMS browser v4.2.

## Figures and Tables

**Table 1:** Data Collection, Phasing, and Refinement Statistics

Crystal Form	Lysenin-Se-Met	Lysenin-Hg^a^	Lysenin:SM	Native Lysenin
Space group	P6_5_22	P6_5_22	C2	P1
Unit cell dimensions				
a, b, c (Å)	98.1, 98.1, 184.172	98.43, 98.43, 184.68	211.26, 37.22, 96.79	58.91, 85.56, 108.81
α, β, γ	90, 90, 120	90, 90, 120	90.0, 104.02, 90.0	98.877, 96.845, 90.036
Resolution (Å)	32.11–2.84 (2.92–2.84)	33.59–2.54 (2.68–2.54)	62.142–3.1	44.36–3.3 (3.48–3.3)
R_merge_	0.104 (0.843)	0.051 (0.561)	0.09 (0.578)	0.159 (0.46)
R_pim_	0.025 (0.155)	0.014 (0.146)	0.055 (0.349)	0.1 (0.291)
I/σ(I)	29.0 (4.9)	33 (5.2)	11.5 (2.2)	8.3 (2.8)
Completeness	99.9 (99.9)	99.3 (98.3)	99.7 (99.5)	97.79 (97.4)
Wave length (Å)	Peak: 0.97867 Remote: 0.9077	1.00941	0.9763	0.9537
No. of sites	7	2		
Phasing power iso^b^ (acentrics/centrics)	Remote: 0.746/0.705 (5.02/7.89)^c^	1.673/1.336 (3.99/5.02)^c^		
Phasing power ano^b^ (acentrics only)	Peak: 1.0 (3.99)^c^ Remote: 0.788 (3.99)^c^	1.09 (3.26)^c^		
Figure of merit (MIRAS) 33.8–2.54 Å	0.46 (acentrics) 0.35 (centrics)			

**Refinement statistics**				

No. of atoms				
Protein	2,355		4,657	9,277
Ligands/ions	44/28		36/15	48/43
Water	24		5	8
Total	2,451		4,713	9,376
Average B-factor (Å^2^)				
Protein	75.7		98.8	68.9
Water	40.5		46.2	17.5
Ligands/ions	75/97		104/125	80.4/80
Resolution (Å)	23.70–2.84		50.38–3.12	29.09–3.3
R_work_/R_free_ (%)	22.35/26.40		24.03/27.07	21.3/23.6
Rmsd				
Bonds (Å)	0.009		0.007	0.07
Angles (°)	1.1		0.94	0.94
Dihedrals (°)	2.97		2.75	2.58
Mol probity score (%)	2.50 (91)		1.54 (100)	1.71 (100)
Ramachandran plot				
Favored	94.22 (%)		93.99 (%)	95.25 (%)
Outliers	0 (%)		0.34 (%)	0.60 (%)

R_merge_ = Σ|I(h,i) − < I(h) > |/ΣI(h,i), where < I(h) > is the mean intensity of reflections. R_pim_ = precision indicating R_merge_ factor. R_work_ and R_free_ were calculated from working and test set reflections. Values for the highest resolution shells are given in parenthesis. Bijvoet pairs are merged.

a N-terminal domain disordered, thus did not carry out refinement.

b Phasing power is the root-mean-square (rms) value of F_h_ divided by the rms lack-of-closure, as given by SHARP. Isomorphous and anomalous difference phasing powers are given, respectively.

c Values in parentheses refer to the resolution at which the Phasing power reaches 1.0.

**Table 2:** Mean Phase Residuals in Resolution Shells for Merged 2D Crystal Images with *p*3 Symmetry

Resolution Range (Å)	No. of Spots	Mean Phase Residual (°)
∞-25	141	27.0
∞-20	209	26.6
∞-15	371	35.9

Four crystal images corresponding to 5,973 unit cells were merged on a common *p*3 phase origin.

## Accession Numbers

The structures described in this manuscript have been deposited in the RCSB PDB with accession codes 3ZX7 (spacegroup P6_5_22), 3ZXD (spacegroup P1), and 3ZXG (spacegroup C2, with sphingomyelin).

## Supplemental Information

Document S1. Figures S1–S5

Movie S1. Conformational Changes Associated with Phosphocholine Binding to the C-Terminal Domain of Lysenin, Related to Figure 3

Movie S2. Conformational Changes Associated with Full-Length Sphingomyelin Binding to the N-Terminal PFM of Lysenin, Related to Figure 3

Movie S3. Cell Permeation by Wild-type Lysenin Monitored by Sytox Green Entry Staining Nucleic Acid Cell Membrane Labeled Using CellMask PM Orange, Related to Figure 4

Movie S4. Cell Permeation by Lys21Ala Lysenin Monitored by Sytox Green Entry Staining Nucleic Acid Cell Membrane Labeled Using CellMask PM Orange, Related to Figure 4

Movie S5. Cell Permeation by Glu128Ala Lysenin Monitored by Sytox Green Entry Staining Nucleic Acid Cell Membrane Labeled Using CellMask PM Orange, Related to Figure 4

Movie S6. Cell Permeation by Gln117Ala Lysenin Monitored by Sytox Green Entry Staining Nucleic Acid Cell Membrane Labeled Using CellMask PM Orange, Related to Figure 4

Movie S7. Cell Permeation by Tyr24Ala Tyr26Ala Lysenin Monitored by Sytox Green Entry Staining Nucleic Acid Cell Membrane Labeled Using CellMask PM Orange, Related to Figure 4
