# Supplementary material for: Structures of Lysenin Reveal a Shared Evolutionary Origin for Pore-Forming Proteins And Its Mode of Sphingomyelin Recognition
Source: Structure. 2012 Sep 5;20(9-3):1498–507. doi: 10.1016/j.str.2012.06.011 (PMC3526787; doi:10.1016/j.str.2012.06.011)
Supplement: Document S1. Figures S1–S5 [file mmc1.pdf]

Structure, Volume *20*

Supplemental Information

Structures of Lysenin Reveal a Shared Evolutionary Origin for Pore-Forming Proteins And Its Mode of Sphingomyelin Recognition

Luigi De Colibus, Andreas F.-P. Sonnen, Keith J. Morris, C. Alistair Siebert, Patrizia Abrusci, Jürgen Plitzko, Vesna Hodnik, Matthias Leippe, Emanuela Volpi, Gregor Anderluh, and Robert J.C. Gilbert

Inventory of Supplemental Information
Figure S1. Surface properties of Lysenin and evolutionarily related toxins.
Relates to Figure 2.

Figure S2. SM electron density map.
Relates to Figure 3.

Figure S3. Fluoresce spectra of WT lysenin and its mutants.
Relates to Figure 4.

Figure S4. Molecular dynamics simulation of wild type and tyrosine double-mutant lysenin.
Relates to Figure 4.

Figure S5. Two-Dimensional crystals of lysenin on liposomes
Relates to Figure 5.

Movie S1: Binding of phosphocholine to C-terminal β-trefoil domain.
Relates to Figure 3.

Movie S2: Binding of sphingomyelin to N-terminal PFM domain.
Relates to Figure 3.

Movie S3. cell permeation by wild-type lysenin monitored by sytox green entry staining nucleic acid cell membrane labeled using cellmask pm orange.
Relates to Figure 4.

Movie S4. cell permeation by lys21ala lysenin monitored by sytox green entry staining nucleic acid cell membrane labeled using cellmask pm orange.
Relates to Figure 4.

Movie S5. cell permeation by glu128ala lysenin monitored by sytox green entry staining nucleic acid cell membrane labeled using cellmask pm orange.
Relates to Figure 4.

Movie S6. cell permeation by gln117ala lysenin monitored by sytox green entry staining nucleic acid cell membrane labeled using cellmask pm orange.
Relates to Figure 4.

Movie S7. cell permeation by tyr24ala tyr26ala lysenin monitored by sytox green entry staining nucleic acid cell membrane labeled using cellmask pm orange.
Relates to Figure 4.

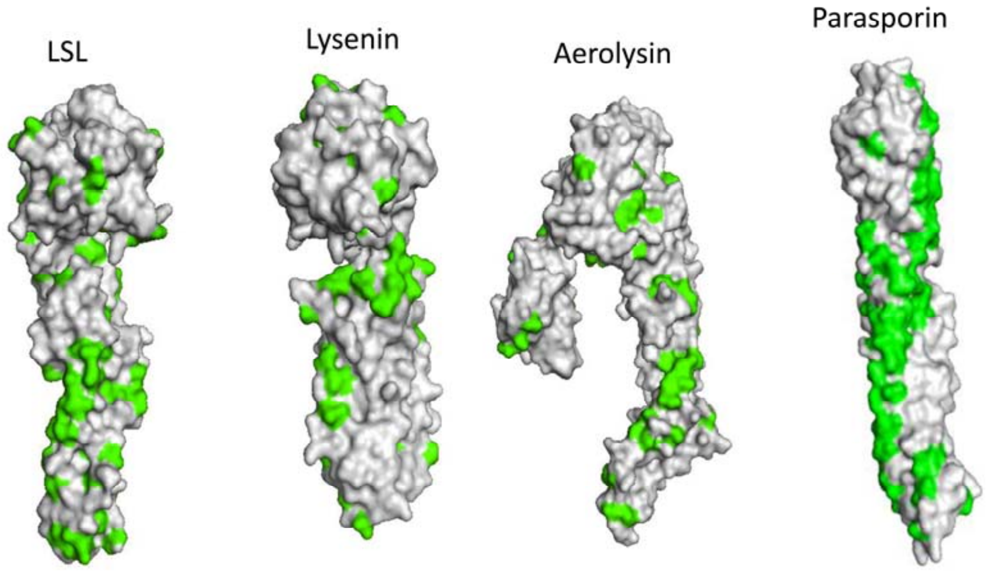

Figure S1, related to Figure 2. Surface properties of Lysenin and evolutionarily related toxins.

Surface representations to show the serine and threonine distribution (colored in green) within the structures.

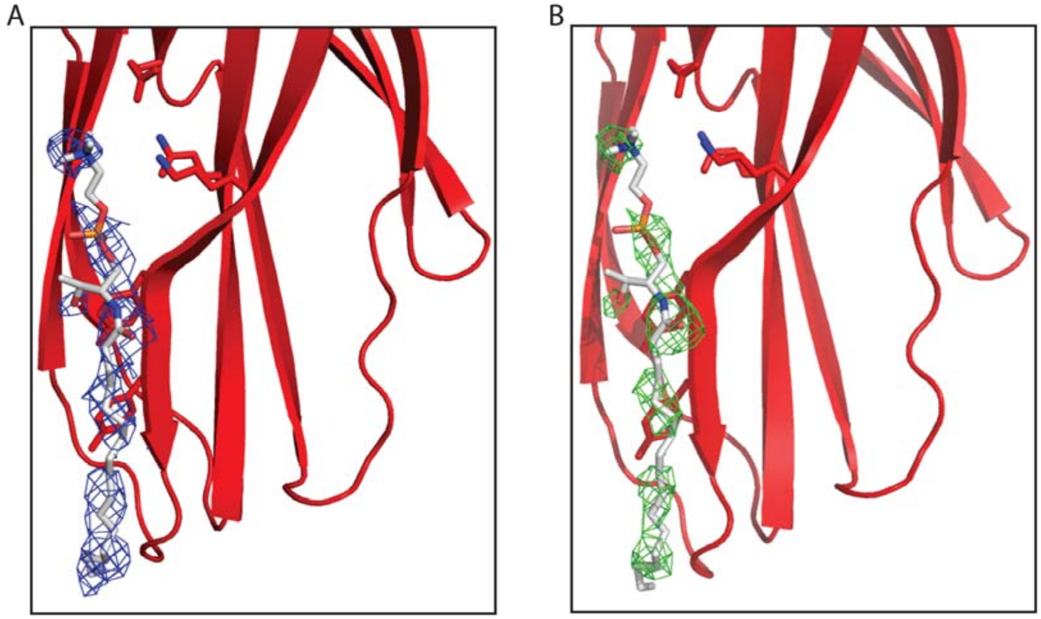

Figure S2, related to Figure 3. SM electron density map.

An omit map was calculated by removing the ligand from the model, then subjecting the model to a 2000K simulated annealing run followed by positional and B factor refinement. The resulting 2Fo-Fc (A) and Fo-Fc (B) maps are contoured respectively at 1.0 and 2.5 σ.

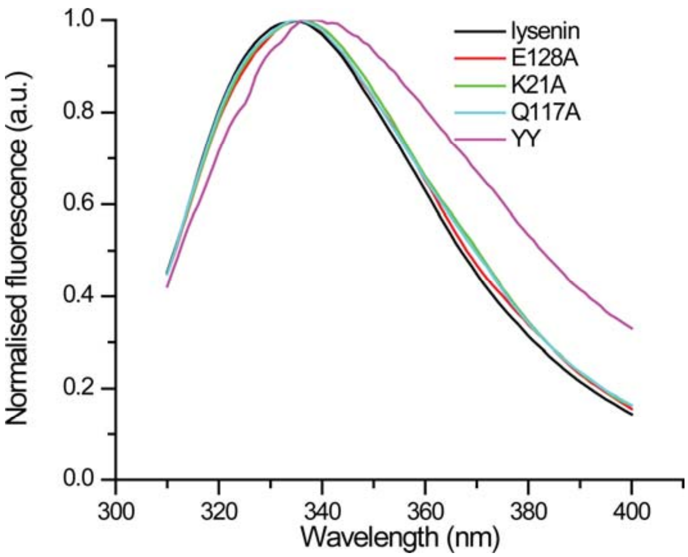

Figure S3, related to Figure 4. Fluoresce spectra of WT lysenin and its mutants.

Fluorescence emission plots of the lysenin WT, single mutants K21A, E128A, Q117A, and double mutant Y24A-Y26A.

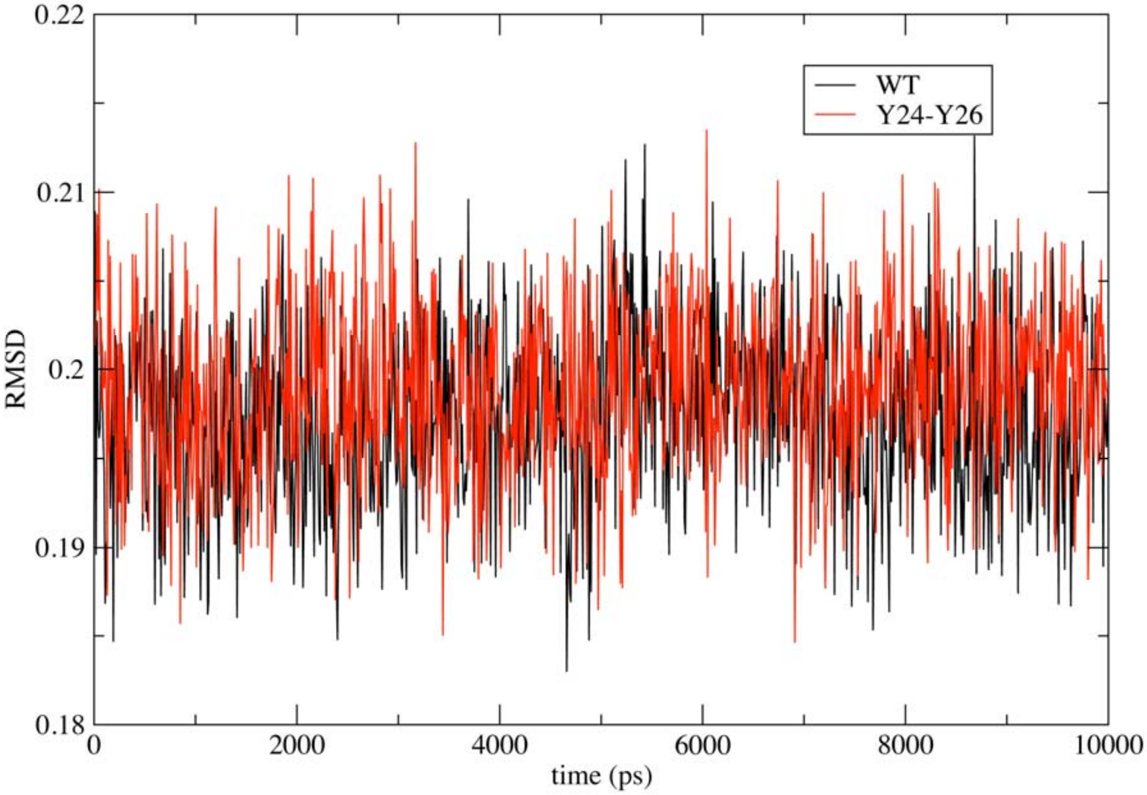

Figure S4, related to Figure 4. Molecular dynamics simulation of wild type and tyrosine double-mutant lysenin.

RMSD fluctuation of the wild type (black) and Tyr24Ala-Tyr26Ala mutant (red) lysenin over 10 ns. Both the wild type and mutant structures show the same fluctuation amplitude.

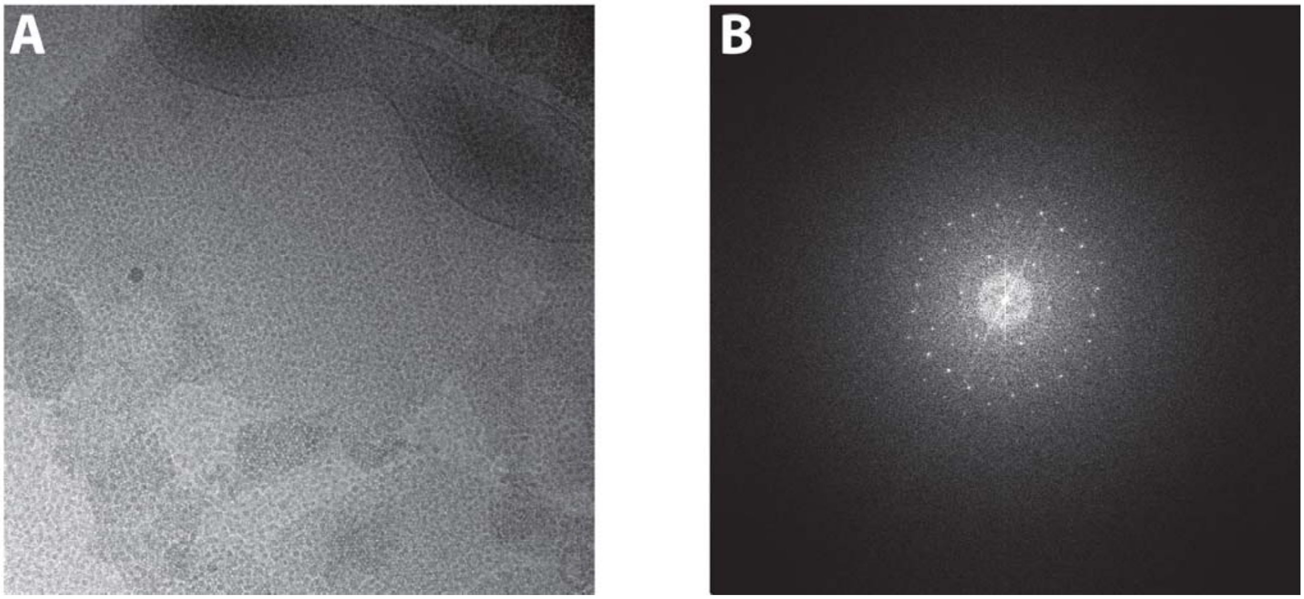

Figure S5, related to Figure 5. Two-Dimensional crystals of lysenin on liposomes

A) Lysenin 2D crystal showing the honeycomb structure.

B) Diffraction pattern for the honeycomb shown in Figure S4A.
